# Association between antibiotics and treatment efficacy in metastatic urothelial carcinoma patients

**DOI:** 10.1186/s12916-024-03786-1

**Published:** 2025-02-25

**Authors:** Avery Braun, Mengying Deng, Jill S. Hasler, Laura Bukavina, Elizabeth Handorf, Philip H. Abbosh

**Affiliations:** 1 Department of Urologic Surgery, University of California Davis, 4860 Y Street, Suite 3500, Sacramento, CA 95817 USA; 2 Biostatistics and Bioinformatics, Fox Chase Cancer Center, Philadelphia, PA 19111 USA; 3 Nuclear Dynamics and Cancer Program, Fox Chase Cancer Center, Philadelphia, PA 19111 USA; 4 Department of Urology, University Hospitals Cleveland Medical Center, Case Western Reserve School of Medicine, Cleveland, OH 44106 USA; 5 Department of Urology, Einstein Healthcare Network, Philadelphia, PA 19141 USA

**Keywords:** Metastatic urothelial carcinoma, Antibiotic therapy, Immune checkpoint inhibitors, Cisplatin-based chemotherapy, Treatment efficacy

## Abstract

**Background:**

Antibiotic therapy (ABT)-induced dysbiosis may affect the efficacy of immune checkpoint inhibitors (ICI) therapy. We investigated the association between ABT and real-world overall survival (rwOS) and progression-free survival (rwPFS) in patients with metastatic urothelial carcinoma (mUC) receiving ICI or cisplatin-based chemotherapy (CIS).

**Methods:**

Three thousand, one hundred seventy-nine patients were included from a nationwide electronic health record-derived de-identified database. Three-month landmark Kaplan–Meier methods and log-rank tests were used to estimate rwOS/PFS between treatment modalities based on ABT groups (stratified by exposure, timing, excretion mode, and administration route). Cox proportional models with time-varying coefficients were used to investigate the associations between ABT, treatment modality, and rwOS/PFS.

**Results:**

A total of 402 (27.1%) ICI and 655 (38.6%) CIS patients received ABT (*p* < 0.001). ICI receipt (OR 0.65, *p* < 0.001) and advanced age (OR 0.98, *p* < 0.001) were associated with lower ABT use.

ICI exclusive findings included a negative correlation with rwOS in patients who received post-treatment initiated (ICI median: pre—13.2 vs post—7.9 vs none—13.3 months; *p* = 0.009), oral (median oral—9.6 vs none—13.3 months, *p* = 0.03), and renally cleared (median renal—9.9 vs none—13.3 months, *p* = 0.04) ABT.

ABT’s effect was negatively associated with rwOS in ICI patients within first 6 months (HR 1.36, 95% CI 1.107–1.74, *p* = 0.01) but not thereafter (*p* = 0.7).

**Conclusions:**

This study identified a potential ICI-specific negative correlation between ABT and rwOS in patients with mUC, specifically those exposed to ABT pills and receipt before treatment initiation. These results emphasize the importance of antibiotic stewardship and continued investigation of the role of gut microbiome in mUC treatment efficacy.

**Supplementary Information:**

The online version contains supplementary material available at 10.1186/s12916-024-03786-1.

## Background

The first-line treatment for metastatic urothelial carcinoma (mUC) has been cisplatin-based chemotherapy (CIS) for nearly three decades. However, the 5-year survival rates remain around 15% in those able to receive CIS [1]. Introduction of immune checkpoint inhibitors (ICIs) following KEYNOTE-052 [2], and IMvigor210 [3], has revolutionized mUC management with guidelines recommending first-line ICI maintenance therapy following chemotherapy in cisplatin-eligible patients [4, 5]. Although ICI therapy is efficacious with a more favorable toxicity profile, the response rates are heterogenous [6], and there are ongoing efforts to identify prognostic or predictive biomarkers along with modifiable factors influencing therapeutic responsiveness [7, 8].

A growing body of literature has facilitated our understanding of the role of the microbiome (urinary and gut) as an essential regulator of the immune system. While the urinary microbiome has demonstrated influence over the tumor micro-environment in non-muscle and muscle invasive localized UC [9], its impact on metastatic disease is less established. However, the gut microbiome (GM) has been indicated as driver of primary resistance to systemic therapies in the metastatic setting [10–12]. In multiple malignancies, the GM demonstrates significant influence over the ability to restore anti-tumor activity of immune cells and mount an adequate therapeutic responsiveness to a multitude of systemic therapies [13–17]. Disturbances to GM homeostasis impact therapeutic responsiveness and investigations have identified antibiotic therapy (ABT) as one mechanism responsible for reduced treatment effectiveness through induction of dysbiosis [11, 15–17].

The landscape of treatment-naive mUC is shifting with evidence from EV-302 supporting first-line enfortumab vedotin-ejfv (EV) and pembrolizumab combination, further establishing therapies targeting the PD1/PDL-1 axis like pembrolizumab as a mainstay of management in this space [18]. Thus, efforts to enhance the efficacy of these agents through GM modulation have been an area of interest [7, 8, 14]. However, within the mUC space, there are a limited studies that have assessed any differential oncologic outcomes related to ABT exposure in patients receiving either ICI or CIS. Utilizing a nationwide electronic health record (EHR)-derived de-identified database, we sought to assess real-world outcomes by investigating the association between ABT and real-world overall survival (rwOS) and progression-free survival (rwPFS) in patients with mUC treated with ICI compared to our control group managed with CIS. Determining causal inference surrounding ABT-induced dysbiosis and efficacy of systemic therapy is a priority, and while prospective randomized control trials are the gold standard, investigating the underlying mechanisms in this setting may raise ethical questions including feasibility of withholding indicated ABT for patients or administering a drug that might negatively impact outcomes. Thus, observational studies describing correlative associations help lay groundwork in uncovering a more nuanced understanding of a potential ABT-GM-ICI interplay. As such, we sought to evaluate the impact ABT exposure, timing of receipt, mode of excretion, and administration route had on patients with mUC receiving ICI and CIS. We hypothesized that ICI recipients exposed to ABT would demonstrate a more pronounced negative association with rwOS/PFS compared to CIS patients.

## Methods

In this retrospective, real-world outcomes analysis, we queried the Flatiron Health (FH) database following Institutional Review Board approval. The FH database comprises de-identified patient-level structured and unstructured data, curated via technology-enabled abstraction originating from 280 US cancer clinics (~ 800 sites of care) [19, 20]. The study queried 10,352 patients with mUC of the bladder or upper tract diagnosed who were treatment-naïve receiving first-line therapy from 01 January 1982 to 14 September 2021 based on abstraction confirmed ICD9/10 codes: ICD-9 188x, 189.1, 189.2, 189.3, or ICD-10 C65x, C66x, C67x, C68.0. Based on abstraction methodology, exclusion of variant histology or stratifying individualized metastatic disease burden was not trackable. Baseline patient (age, sex, race/ethnicity, body mass index [BMI], Eastern Cooperative Oncology Group [ECOG] status, insurance status, history of smoking) and clinical (stage, surgery status) variables were collected along with ABT characteristics. ICI stratification required receipt of any immunotherapeutic agent, whereas chemotherapy stratification was based on receiving CIS. This study analyzed outcomes in the first-line setting pre-dating the approval of EV-pembrolizumab in this space and excluded patients who received carboplatin given known inferior survival benefit compared to CIS [21]. The systemic therapy regimen and ABT classes are listed in Additional file 1: Tab. S1.

Baseline patient characteristics were compared by treatment modality and ABT exposure using *t*-tests and chi-square tests. Multivariable logistic regression models assessed the association between treatment modality and ABT exposure, adjusting for pre-selected covariates. rwOS/PFS were calculated from the date of treatment initiation to the date of real-world progression/date of death and censored at the date of last confirmed activity for OS and last clinic note date for PFS. We used a 3-month landmark for survival analyses to address immortal time bias as ABT use is a time-varying exposure [22]. Three-month landmark Kaplan–Meier and log-rank tests were used to compare oncologic outcomes between ICI and CIS cohorts based on (1) exposure, (2) timing, (3) excretion mode, and (4) administration route. Exposure was categorized as either ABT or no ABT. Timing of exposure was stratified into three windows: no ABT, within the 3 months immediately prior to treatment initiation (pABT), or within the 3 months immediately following treatment initiation to 3 months (referred to as concurrent ABT, cABT). Excretion was categorized as no ABT, hepatic/mixed, or renal. Administration was classified as no ABT, oral (PO), or parenteral (IV) based on extrapolated structured data and confirmation of route of administration using guidetopharmacology.org. Multivariable Cox proportional models were used to investigate the association between rwOS/PFS, ABT, and treatment modality after adjusting for covariates. The interaction effect between the treatment groups and ABT was measured to ascertain whether the effect of ABT differed among treatments. We included a time-varying effect of ABT (split between the first 6 months and subsequent follow up) when non-proportional hazards were detected. Statistical significance was set at a *p*-value ≤ 0.05. R version 4.3.0 was used on all analyses.

## Results

### Demographics and treatment modality

A total of 3179 patients with mUC (Additional file 1: Fig. S1) were included with baseline demographic and clinical characteristics described in Table 1. A total of 1483 (46.6%) patients received ICI (median age 76.9 years, IQR 69.8–81.3), and 1696 patients (53.4%) received CIS (median age 68.2 years, IQR 61.7–73.8). The ICI group was older (76.8 vs 71.5, *p* < 0.001), had higher ECOG status (*p* < 0.001), and was less likely to have undergone surgery after advanced diagnosis (21.8% vs 4.2%, *p* < 0.001). There was no evidence of difference between two treatment groups by race/ethnicity (*p* = 0.2) and sex (*p* = 0.3). More patients in the CIS group received ABT (39% vs 27%, *p* < 0.001). Between groups, there was a significant unadjusted difference in rwOS with CIS faring better (median 15.6 vs 19.2 months, *p* < 0.001) but no difference in rwPFS (median rwPFS 9.47 vs 9.41 months, *p* = 1). During the study period, a total of 1055 CIS and 1004 ICI patients experienced a death event while 1314 CIS and 1184 ICI patients had progression or death event.

**Table 1:** Patient characteristics by first-line treatment modality

	CIS (*N* = 1696)	ICI (*N* = 1483)	Total (*N* = 3179)	*p* value
Age at diagnosis				< 0.001^a^
Mean (SD)	67.3 (8.7)	74.7 (8.2)	71.3 (8.7)	
Median	68.2	76.9	72.9	
Range	31.6–85.1	33.7–85.1	23.1–85.2	
Q1, Q3	61.8, 73.8	69.8, 81.3	65.8, 77.9	
Sex (%)				0.3^b^
Female	456 (27)	424 (29)	880 (28)	
Male	1240 (73)	1059 (71)	2299 (72)	
Stage at diagnosis (%)				< 0.001^b^
I–III	251 (15)	399 (27)	650 (20)	
IV	785 (46)	361 (24)	1146 (36)	
Unknown/other	660 (39)	723 (49)	1383 (44)	
Race (%)				0.2^b^
White	1180 (70)	1038 (70)	2218 (70)	
Asian	26 (1.5)	17 (1.1)	43 (1.4)	
Black or African American	79 (4.7)	51 (3.4)	130 (4.1)	
Hispanic or Latino	67 (4.0)	48 (3.2)	115 (3.6)	
Other	227 (13)	209 (14)	436 (14)	
Unknown	117 (6.9)	120 (8.1)	237 (7.5)	
Insurance type (%)				< 0.001^b^
Commercial	565 (33)	387 (26)	952 (30)	
Medicaid	119 (70)	77 (5.2)	196 (6.2)	
Medicare	299 (18)	338 (23)	637 (20)	
Medicare/commercial	473 (28)	489 (33)	962 (30)	
Other	147 (8.7)	144 (9.7)	291 (9.2)	
Unknown	93 (5.5)	48 (3.2)	141 (4.4)	
Surgery after diagnosis (%)				< 0.001^b^
No	1326 (78)	1420 (96)	2746 (86)	
Yes	370 (22)	63 (4.2)	2627 (13.6)	
ECOG status (%)				< 0.001^b^
0	580 (34)	325 (22)	905 (29)	
1	486 (29)	512 (35)	998 (31)	
2	89 (5.2)	179 (10)	311 (9.8)	
3	18 (1.1)	78 (5.3)	96 (3.0	
4	0 (0.0)	2 (0.1)	2 (0.1)	
Unknown	532 (31)	344 (23)	867 (27)	
Metastasis within 1 month of diagnosis (%)				< 0.001^b^
No	665 (39)	1068 (72)	1733 (55)	
Yes	1031 (61)	415 (28)	1446 (45)	
Antibiotic exposure (%)				< 0.001^b^
No	1041 (61)	1081 (73)	2122 (67)	
Yes	655 (39)	402 (27)	1057 (33)	

Multivariable analysis 3 months landmark overall survival and real-world progression-free survival with time-varying coefficient, adjusting for covariates (age, sex, body mass index, smoking status, surgery receipt, race, stage at diagnosis, insurance type, ECOG status)

*ECOG* Eastern Cooperative Oncology Group Performance Status Scale

^a^Linear model ANOVA

^b^Pearson’s chi-squared test

### Demographics and antibiotic exposure

A total of 1057 (33%) patients were exposed to ABT who tended to be younger (69.4 vs 71.4, *p* < 0.001) and female (31% vs 26%, *p* = 0.005). There was a significant difference in ABT exposure by race (73% vs 68% white, *p* < 0.001), ECOG status (ECOG 0 27% vs 29%, *p* < 0.001), but not receipt of surgery after metastatic diagnosis (surgical recipient 15% vs 13%, *p* = 0.1; Additional file 1: Tab. S2). In multivariable analysis, race, ECOG status, smoking history, BMI, and stage at diagnosis were not associated with ABT exposure. However, ICI therapy (OR 0.65, 95% CI 0.54–0.78, *p* < 0.001), advanced age per 10 years (OR 0.82, 95% CI 0.74–0.90, *p* < 0.001), and male sex (OR 0.78, 95% CI 0.66–0.93, *p* = 0.005) were associated with lower ABT use (Additional file 1: Tab. S3). Among ABT recipients, there was a total of 704 death events and 855 progression or death events; in those who did not receive ABT, there were 1355 death events and 1643 progression or death events.

### ABT exposure and timing

**Fig. 1:**
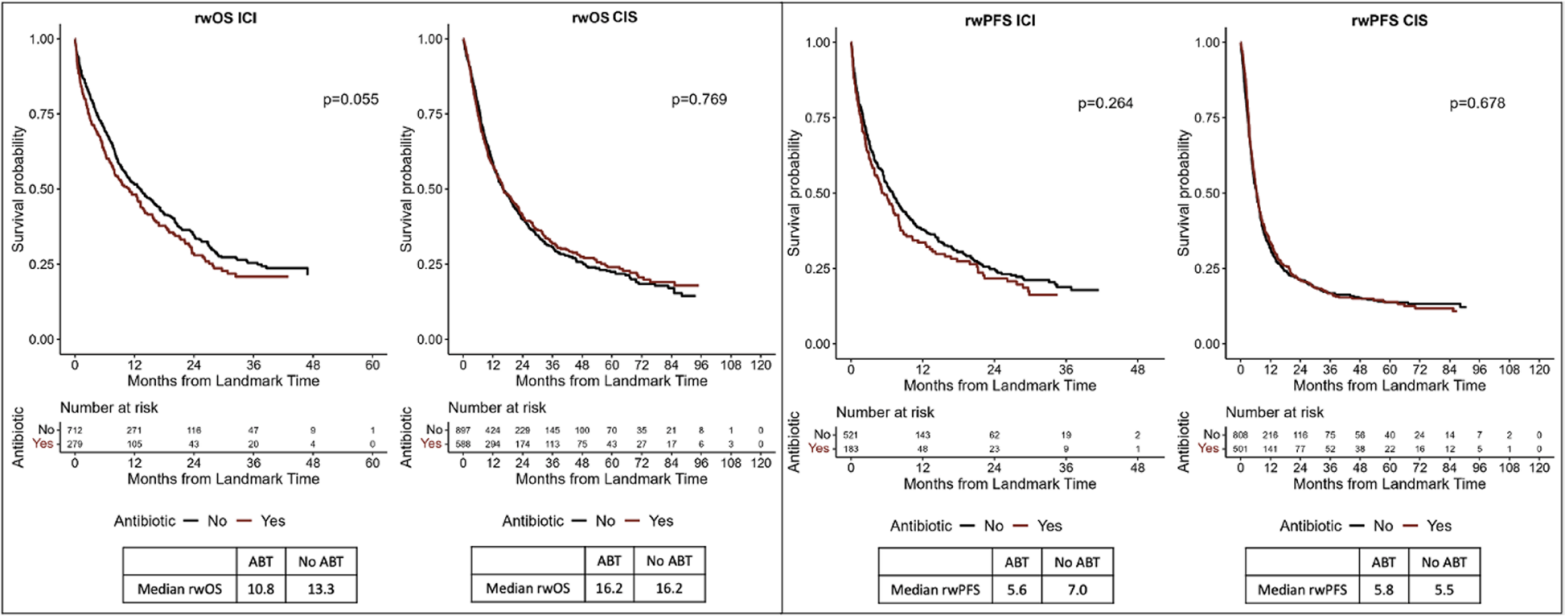
Oncologic outcomes by antibiotic therapy exposure. **A** Overall survival by antibiotic exposure. **B** Real-world progression-free survival by antibiotic exposure. OS, overall survival; rwPFS, real-world progression-free survival; ICI, immunotherapy; non-ICI, chemotherapy. 3-month landmark Kaplan–Meier (KM) curves

We hypothesized that ABT exposure would have a negative correlation with rwOS/PFS in patients receiving ABT prior to starting ICI therapy compared to those not receiving ABT or receiving ABT after ICI initiation. ABT exposure (any ABT vs no ABT) was not significantly associated with rwOS or rwPFS in ICI (rwOS median 10.8 vs 13.3 months, *p* = 0.06; rwPFS median 5.6 vs 7.0, *p* = 0.3) or CIS (OS. median 16.2 vs 16.2, *p* = 0.8; rwPFS median 5.8 vs 5.5, *p* = 0.7) cohorts (Fig. 1).

**Fig. 2:**
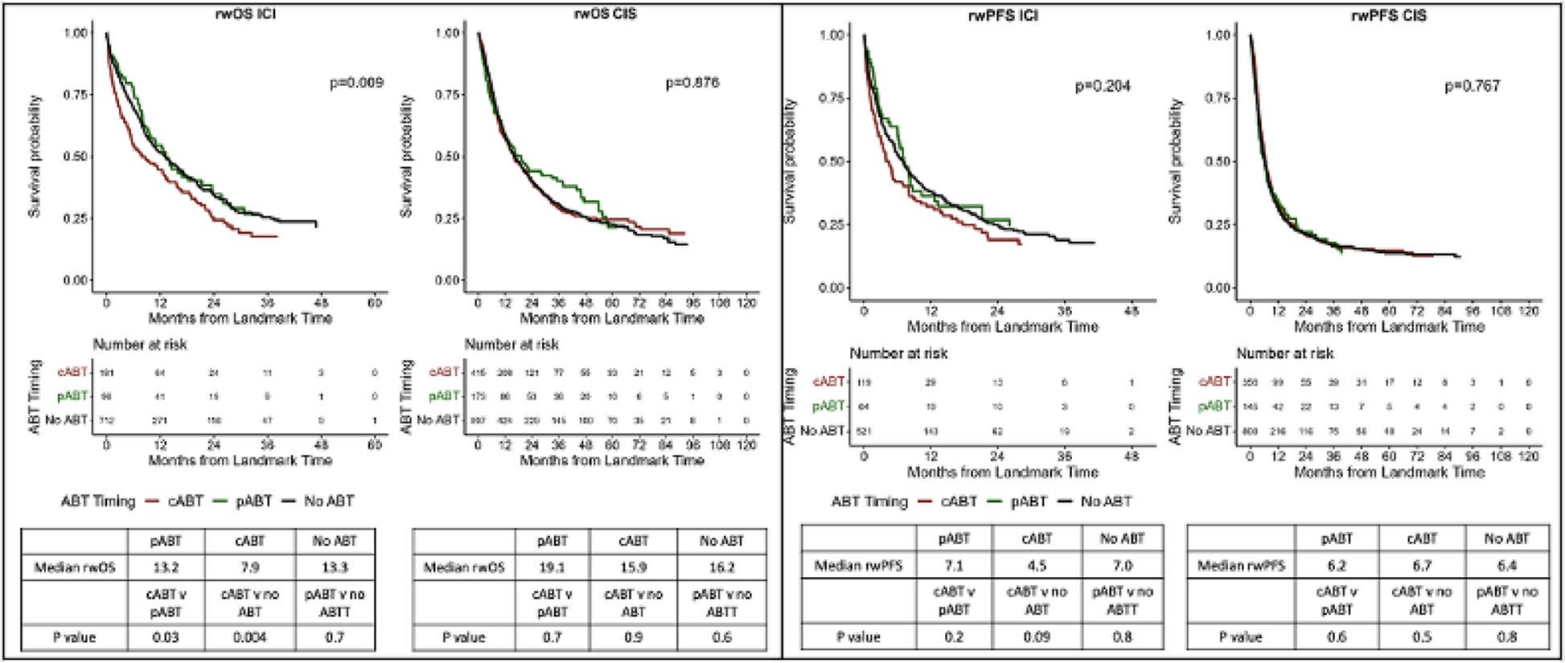
Oncologic outcomes by antibiotic timing with pairwise comparisons. **A** Overall survival by antibiotic timing. **B** Real-world progression-free survival by antibiotic timing. OS, overall survival; rwPFS, real-world progression-free survival; ICI, immunotherapy; non-ICI, chemotherapy; ABT, antibiotic therapy; pABT, before treatment initiation, cABT, concurrent or after treatment initiation. 3-month landmark Kaplan–Meier (KM) curves; median survival with pairwise comparisons

However, timing of ABT exposure was negatively associated with rwOS in the ICI group (median 7.9 months cABT vs 13.2 pABT vs 13.3 no ABT, *p* = 0.009) but not in the CIS group (*p* = 0.9). In pairwise comparison, there was a negative association with rwOS in ICI patients who received ABT after starting therapy compared to prior to treatment initiation (*p* = 0.03) as well as compared to no ABT (*p* = 0.004). There was no association between rwPFS and ABT timing either treatment group (Fig. 2).

### Association of ABT excretion mode

**Fig. 3:**
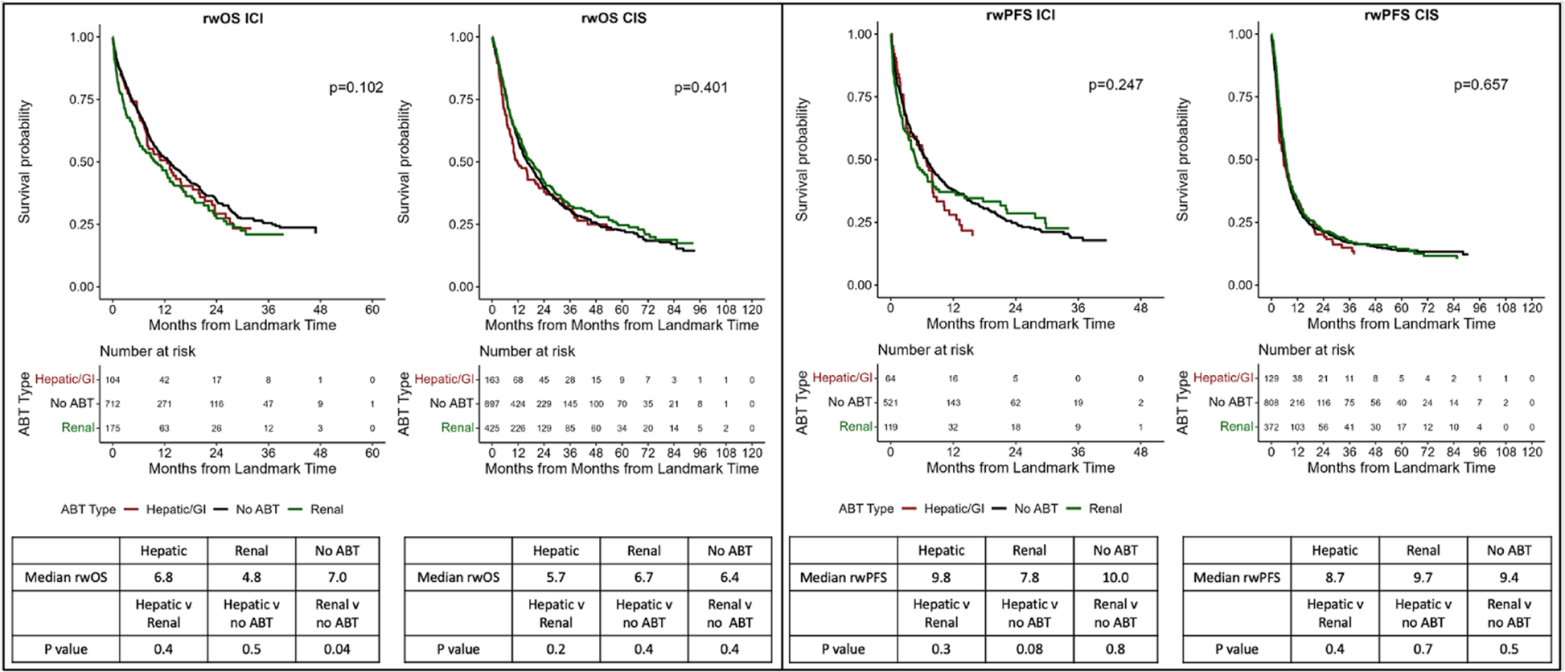
Oncologic outcomes by antibiotic therapy excretion mode with pairwise comparisons. **A** Overall survival by antibiotic excretion. **B** Real-world progression-free survival by antibiotic excretion. OS, overall survival; rwPFS, real-world progression-free survival; ICI, immunotherapy; non-ICI, chemotherapy; ABT, antibiotic therapy. 3-month landmark Kaplan–Meier (KM) curves; median survival with pairwise comparisons

We hypothesized that hepatically excreted ABT would be negatively correlated with rwOS compared to other modes of excretion. rwOS was not statistically significantly associated with ABT excretion mode (hepatic/mixed vs renal vs none) for ICI (*p* = 0.1) or CIS (*p* = 0.4). However, in pairwise comparison in ICI patients, receipt of renally cleared ABT was negatively associated with rwOS (median rwOS 4.8 vs 7.0 months, *p* = 0.04) compared to those with no ABT. No correlative association was identified between excretion mode and rwPFS in either group (ICI: *p* = 0.3; CIS: *p* = 0.7), and pairwise comparison did not demonstrate any associations (Fig. 3).

### Association of ABT administration route

**Fig. 4:**
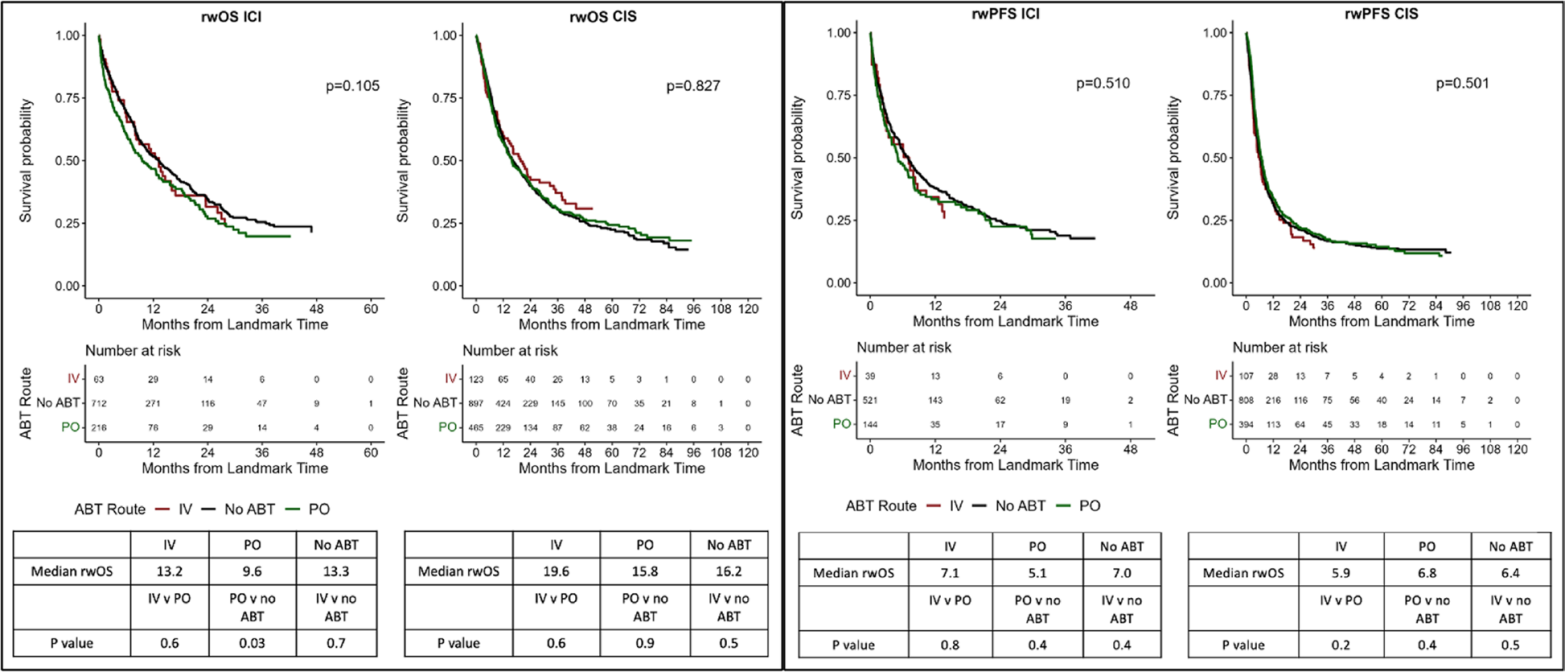
Oncologic outcomes by antibiotic therapy administration roue with pairwise comparisons. **A** Overall survival by antibiotic administration. **B** Real-world progression-free survival by antibiotic administration. OS, overall survival; rwPFS, real-world progression-free survival; ICI, immunotherapy; non-ICI, chemotherapy; ABT, antibiotic therapy; IV, intravenous; PO, oral. 3-month landmark Kaplan–Meier (KM) curves; median survival with pairwise comparisons

Patients who receive ABT presumably have infections or are presumed to have infections and therefore may be more mortality-prone; however, determining the treatment indication or its severity is not possible within the FH database. However, patients receiving IV ABT are likely to be hospitalized with more severe infection or suspected infection and are likely to be at risk for death. To determine whether such infections might be a confounder, we examined the association between the route of ABT administration and rwOS/PFS. In analyzing the impact of the ABT administration route, no statistically significant correlative association was noted between IV and PO vs no ABT groups for rwOS (ICI: *p* = 0.1, CIS: *p* = 0.8) or rwPFS (ICI: *p* = 0.5; chemotherapy: *p* = 0.8). However, in pairwise comparison of ICI patients, receipt of oral ABT was negatively associated with rwOS compared to no ABT (median 12.6 vs 16.3 months, *p* = 0.034; CIS patients, *p* = 0.9; Fig. 4).

### Cox regression models with time varying coefficients

The coefficients for Cox proportional hazards models for rwOS and rwPFS are listed in Additional File 1: Tab S4-5. These models used a 3-month landmark time-point and time-varying coefficients to account for non-proportional hazards. A 9-month threshold was based on violation of Schoenfeld residuals but corresponds with timeframe for re-establishing GM homeostasis after ABT ranges (3–12 months) [23] and median survival of mUC in first-line setting with current standard of care management (7–15 months) [2, 24]. In the first 6 months following time 0 (i.e., 3 months after the initiation of systemic therapy), a negative correlation with rwOS was seen exclusively in ICI patients (HR 1.36, 95% CI 1.07–1.74, *p* = 0.012) and not in CIS patients (*p* = 0.130); however, the relationship between ABT, ICI, and rwOS did not persist beyond 9 months (*p* = 0.705). Both before and after 6 months, ABT exposure was not associated with rwPFS in either treatment group (Table 2).

**Table 2:** Three-month landmark overall survival and real-world progression-free survival with time-varying coefficients

	Hazard ratio	Lower confidence interval	Higher confidence interval	*p* value
**Overall survival with time-varying coefficients**				
Antibiotic effect in ICI in first 9 months	1.36	1.07	1.74	0.01
Antibiotic effect in ICI after 9 months	1.05	0.81	1.36	0.7
Antibiotic effect in CIS in first 9 months	1.18	0.95	1.47	0.1
Antibiotic effect in CIS after 9 months	0.90	0.76	1.07	0.2
**Real world progression free survival with time-varying coefficients**				
Antibiotic effect in ICI in first 9 months	1.00	0.57	1.76	1
Antibiotic effect in ICI after 9 months	1.08	0.77	1.53	0.7
Antibiotic effect in CIS in first 9 months	0.97	0.82	1.14	0.7
Antibiotic effect in CIS after 9 months	1.03	0.84	1.26	0.8

Multivariable analysis 3-month landmark real world overall survival and progression free survival with time-varying coefficient, adjusting for covariates (rwOS: age, surgery receipt, race, insurance type, ECOG status; rwPFS: surgery receipt, ECOG status)

## Discussion

In our real-world analysis study, we identified an ICI-exclusive negative association between ABT and rwOS in patients with mUC. Overall, ICI patients were older but less likely to receive ABT than CIS patients. Unique to the ICI recipients, those exposed to oral ABT and cABT compared to those without ABT exposure demonstrated a more pronounced negative association with rwOS. This may be due to a more direct effect on the GM. However, the potential effect of ABT on ICI patients’ outcomes appears to be short-lived, as a significant association beyond 6 months after treatment initiation was not detected. Our findings highlight the importance of providers practicing appropriate antibiotic stewardship, particularly for patients with mUC eligible for ICI therapy during their early treatment course.

With evidence from EV-302, pembrolizumab—a programmed cell death protein 1 (PD-1) inhibitor—is a pivotal agent in management of mUC in the first-line setting [18]. Thus, our finding that ABT exposure may disproportionally impact patients with mUC receiving ICI is important to acknowledge and might be rooted in disease pathophysiology. UC tumor microenvironments highly express programmed death-ligand 1 (PD-L1), and trials such as IMvigor2019 and KEYNOTE-052 have reported blunted responses to ICI in individuals with low PD-L1 expression [2, 3]. With knowledge that therapeutic responsiveness in UC may be linked to PD-L1 expression, Sivan et al. assessed the impact of manipulating the GM to modulate PD-L1 cancer immunotherapy [11]. They found that mice with melanoma exposed to anti-PD-L1 therapy plus a favorable fecal microbiome transplant (FMT) experienced improved tumor control compared to either monotherapy arm. Routy et al. then confirmed the negative influence of ABT exposure on the GM-ICI axis through their work with ABT-treated mice, demonstrating restoration of anti-PD-1 therapy responsiveness following FMT from PD-1 responsive cancer patients [15]. These pivotal translational studies laid important groundwork in understanding the critical association between the ABT-induced dysbiosis and ICI responsiveness.

Given the increased use of ICI for the management of mUC, efforts to understand the nuanced relationship between ABT, dysbiosis, and ICI efficacy are ongoing. Upwards of twenty meta-analyses are published on ABT and ICI interaction with the most recent and comprehensive including 5454 patients with mUC [25]. A significant reduction in OS but not PFS was noted in mUC patients and most prominent in those who received ABT within 60 days before or after treatment initiation. However, granular elucidation of the timeframe of exposure is strongly associated with reduced survival in mUC patients has produced conflicting results; Pinato et al. found that pABT was associated with worsened outcomes [26], while robust post hoc analysis from IMvigor210 and IMvigor211 trials found an ICI-exclusive negative association with worse OS from cABT exposure [27]. While our findings align with the growing consensus that ABT is associated with reduced OS in patients with mUC on ICI, we sought to go beyond ABT timing and evaluate a fuller breadth of characteristics. Our study is distinguished from these other studies by the analysis of a contemporaneous additional control group, patients receiving CIS. Our findings that most of the significant associations of ABT use with survival were specific to the ICI group strongly support a role for the negative impact of ABT in cancer patients receiving ICI.

Across malignancies, a higher cumulative ABT treatment duration [28] and broader spectrum ABT [29] have been associated with worse outcomes but less is known definitively about excretion and administration. The effect of clearance mode and administration route on survival in ICI patients is unknown; therefore, we stratified these parameters and found that renal clearance and PO administration are negatively associated with survival in patients receiving ICI. It is theorized that clearance route impacts the magnitude with which ABT alters GM through the microbiome-liver axis [30]; this axis facilitates crosstalk of hepatically excreted ABT and the gut microenvironment modulating the GM to a higher degree than those renally cleared [31]. Our hypothesis that hepatically cleared ABT would be independently associated with reduced rwOS/PFS, however, was not supported; it can be speculated that excretion mode may play a lesser role in influencing outcomes as mouse-models have demonstrated changes in the GM’s biodiversity and composition of commensal bacteria from ABT both hepatically or renally cleared [32]. Moreover, the degree to which ABT administration route contributes to GM perturbations is debated, with conflicting evidence on the differential effects oral and parenteral delivery has. It has been theorized that oral ABT inevitably induces a higher degree of dysbiosis [33] because it interacts more directly with the GM, with other studies arguing that parenteral delivery minimally alters the gut or facilitates a quicker return to pre-ABT GM richness and diversity than the oral route [34].

There are limitations to this retrospective study, particularly in ascertaining if other comorbidities influenced ABT use in patients with concomitant medical conditions and thus exerted a compounded effect on outcomes independent of ABT. FH lacks granular information regarding metastatic disease burden as well as the setting of antibiotic administration (inpatient vs outpatient), dose duration (acute vs chronic), its implications on indication (prophylactic vs therapeutic), and severity of infection, all of which could independently drive poor survival outcomes. However, we attempted to limit unmeasured confounding factors by assessing the administrative route, an indirect measure of setting, and severity of infection. FH collects data through electronic health records, which may limit the capture of all ABT prescriptions if written outside of the network or not self-reported by patients. Moreover, the impact of individualized diet on the GM cannot be accounted for due to the retrospective nature of this work. Finally, our study did not incorporate the use of biomarkers such as PD-L1, TMB, and T cell infiltration into tumors as diagnostic markers or microbiome analysis (fecal or urinary). Thus, our findings suggest a possible association between ABT exposure, ICI recipient, and worse oncologic outcomes; however, we cannot claim a direct interaction.

Our findings that oral ABT during ICI treatment was associated with worse OS is notable for two key reasons. First, this occurred in a cohort of patients who were younger with less advanced disease at diagnosis and were deemed appropriate surgical candidates compared to the chemotherapy reference group. Second, with the approval of neoadjuvant ICI on the horizon for localized muscle-invasive UC and initial studies demonstrating similar associations between cABT and outcomes as found in mUC [35], it is an imperative to fully understand the intricacies of the ABT-GM-ICI relationship to provide the best care for patients throughout their disease course, localized to metastatic.

## Conclusion

This study identifies a potential negative association between ABT and rwOS in patients with mUC receiving ICI but not CIS. These results support the relevance of the GM on immunotherapy efficacy and the PD1/PDL-1 axis. Although further studies are needed, our findings emphasize the importance of antibiotic stewardship in improving oncologic outcomes in patients receiving first-line ICI therapy for mUC.

## Supplementary Information

Additional file 1: Figs. S1 and Table S1-S5. Fig. S1: Final cohort CONSORT Diagram. Table S1: List of first-line systemic therapy and antibiotic regimens. Table S2: Patient characteristics by antibiotic receipt. ^a^Linear model ANOVA**; ^b^Pearson’s chi-squared test**. Table S3: Logistic regression to predict antibiotic receipt. ^a^Multivariable logistic regression; ECOG, Eastern Cooperative Oncology Group; ICI, immunotherapy. Table S4: Three-month landmark overall survival with interaction adjusting for time-varying coefficients. ECOG, Eastern Cooperative Oncology Group. Table S5: Three-month landmark progression-free survival with interaction adjusting for time-varying coefficients. ECOG, Eastern Cooperative Oncology Group.

## Data Availability

The data that support the findings of this study are available from the corresponding author upon reasonable request.

## Abbreviations

ABT: Antibiotic therapy
ICI: Immune checkpoint inhibitors
rwOS: Real-world overall survival
rwPFS: Real-world progression-free survival
mUC: Metastatic urothelial carcinoma
CIS: Cisplatin-based chemotherapy
GM: Gut microbiome
EV: Enfortumab vedotin-ejfv
EHR: Electronic medical records
FH: Flatiron Health
BMI: Body mass index
ECOG: Eastern Cooperative Oncology Group
pABT: Prior to treatment initiation antibiotic exposure
cABT: Antibiotic exposure within 3 months immediately following treatment initiation to 3 months
PD-1: Programmed cell death protein 1
PD-L1: Programmed death-ligand 1
FMT: Fecal microbiome transplant

## References

[1] Galsky MD, Hahn NM, Rosenberg J, et al. Treatment of patients with metastatic urothelial cancer “Unfit” for cisplatin-based chemotherapy. J Clin Oncol. 2011;29(17):2432–8. 10.1200/JCO.2011.34.8433. 21555688 10.1200/JCO.2011.34.8433

[2] Balar AV, Castellano D, O’Donnell PH, et al. First-line pembrolizumab in cisplatin-ineligible patients with locally advanced and unresectable or metastatic urothelial cancer (KEYNOTE-052): a multicentre, single-arm, phase 2 study. Lancet Oncol. 2017;18(11):1483–92. 10.1016/S1470-2045(17)30616-2. 28967485 10.1016/S1470-2045(17)30616-2

[3] Balar AV, Galsky MD, Rosenberg JE, et al. Atezolizumab as first-line treatment in cisplatin-ineligible patients with locally advanced and metastatic urothelial carcinoma: a single-arm, multicentre, phase 2 trial. Lancet. 2017;389(10064):67. 27939400 10.1016/S0140-6736(16)32455-2 PMC5568632

[4] Witjes CA, Bruins HM, Cathomas R. EAU Guidelines on muscle-invasive and metastatic bladder cancer. Eur Assoc Urol. 2021:5.2.1.Local staging of MIBC. https://uroweb.org/guideline/bladder-cancer-muscle-invasive-and-metastatic/#5.

[5] Milowsky MI, Bryan Rumble R, Booth CM, et al. Guideline on muscle-invasive and metastatic bladder cancer (European Association of Urology guideline): American Society of Clinical Oncology clinical practice guideline endorsement. J Clin Oncol. 2016;34(16):1945–52. 10.1200/JCO.2015.65.9797. 27001593 10.1200/JCO.2015.65.9797

[6] Lopez-Beltran A, Cimadamore A, Blanca A, et al. Immune checkpoint inhibitors for the treatment of bladder cancer. Cancers (Basel). 2021;13(1):1–16. 10.3390/cancers13010131. 10.3390/cancers13010131 PMC7795541 33401585

[7] Aggen DH, Drake CG. Biomarkers for immunotherapy in bladder cancer: a moving target. J Immunother Cancer. 2017;5(1):1–13. 10.1186/s40425-017-0299-1. 29157296 10.1186/s40425-017-0299-1 PMC5697433

[8] Tripathi A, Plimack ER. Immunotherapy for urothelial carcinoma: current evidence and future directions. Curr Urol Rep. 2018;19(12). 10.1007/s11934-018-0851-7. 10.1007/s11934-018-0851-7 30406502

[9] Andolfi C, Bloodworth JC, Papachristos A, et al. The urinary microbiome and bladder cancer: susceptibility and immune responsiveness. 2020;6(3):225–34. 10.3233/BLC-200277. 10.3233/BLC-200277 PMC7605348 33195783

[10] Sears CL, Pardoll DM. The intestinal microbiome influences checkpoint blockade. Nat Med. 2018;24(3):254–5. 10.1038/nm.4511. 29509750 10.1038/nm.4511 PMC6435254

[11] Sivan A, Corrales L, Hubert N, et al. Commensal Bifidobacterium promotes antitumor immunity and facilitates anti-PD-L1 efficacy. Science (80- ). 2015;350(6264):1084–9. 10.1126/science.aac4255. 10.1126/science.aac4255 PMC4873287 26541606

[12] Derosa L, Zitvogel L. Antibiotics impair immunotherapy for urothelial cancer. Nat Rev Urol. 2020;17(11):605–6. 10.1038/s41585-020-0373-1. 32901131 10.1038/s41585-020-0373-1

[13] Watanabe S, Kikuchi T. Does the gut microbiota play a key role in PD-1/PD-L1 blockade therapy? Transl Lung Cancer Res. 2020;9(3):438–40. 10.21037/tlcr.2020.03.31. 32676307 10.21037/tlcr.2020.03.31 PMC7354115

[14] Wang Y, Ma R, Liu F, Lee SA, Zhang L. Modulation of gut microbiota: a novel paradigm of enhancing the efficacy of programmed death-1 and programmed death ligand-1 blockade therapy. Front Immunol. 2018;9(MAR). 10.3389/fimmu.2018.00374. 10.3389/fimmu.2018.00374 PMC5845387 29556232

[15] Routy B, Le Chatelier E, Derosa L, et al. Gut microbiome influences efficacy of PD-1-based immunotherapy against epithelial tumors. Science (80- ). 2018;359(6371):91–7. 10.1126/science.aan3706. 10.1126/science.aan3706 29097494

[16] Gopalakrishnan V, Helmink BA, Spencer CN, Reuben A, Wargo JA. The Influence of the Gut Microbiome on Cancer, Immunity, and Cancer Immunotherapy. Cancer Cell. 2018;33(4):570–80. 10.1016/j.ccell.2018.03.015. 29634945 10.1016/j.ccell.2018.03.015 PMC6529202

[17] Vétizou M, Pitt JM, Daillère R, et al. Anticancer immunotherapy by CTLA-4 blockade relies on the gut microbiota. Science. 2015;350(6264):1079–84. 10.1126/science.aad1329. 26541610 10.1126/science.aad1329 PMC4721659

[18] Powles T, Valderrama BP, Gupta S, et al. LBA6 EV-302/KEYNOTE-A39: Open-label, randomized phase III study of enfortumab vedotin in combination with pembrolizumab (EV+P) vs chemotherapy 9chemo) in previously untreaed locally advanced metastatic urothelial carcinoma (la/mUC). Ann Oncol. 2023;23:S1340–S1340. 10.1016/j.annonc.2023.10.106.

[19] Birnbaum B, Nussbaum N, Seidl-Rathkopf K, et al. Model-assisted cohort selection with bias analysis for generating large-scale cohorts from the EHR for oncology research. 2020. http://arxiv.org/abs/2001.09765.

[20] Ma X, Long L, Moon S, Adamson B, Baxi SS. Comparison of population characteristics in real-world clinical oncology database in the US: Flatiron Health, SEER and NPCR. medRxiv; 2020;16(March). 10.1101/2020.03.16.20037143.

[21] Galsky MD, Chen GJ, Oh WK, et al. Comparative effectiveness of cisplatin-based and carboplatin-based chemotherapy for treatment of advanced urothelial carcinoma. Ann Oncol. 2012;23(2):406–10. 10.1093/annonc/mdr156. 21543626 10.1093/annonc/mdr156

[22] Suissa S. Immortal time bias in pharmacoepidemiology. Am J Epidemiol. 2008;167(4):492–9. 10.1093/aje/kwm324. 18056625 10.1093/aje/kwm324

[23] Patangia DV, Anthony Ryan C, Dempsey E, Paul Ross R, Stanton C. Impact of antibiotics on the human microbiome and consequences for host health. Microbiologyopen. 2022;11(1): e1260. 10.1002/mbo3.1260. 35212478 10.1002/mbo3.1260 PMC8756738

[24] von der Maase H, Sengelov L, Roberts JT, et al. Long-term survival results of a randomized trial comparing gemcitabine plus cisplatin, with methotrexate, vinblastine, doxorubicin, plus cisplatin in patients with bladder cancer. J Clin Oncol. 2005;23(21):4602–8. 10.1200/JCO.2005.07.757. 16034041 10.1200/JCO.2005.07.757

[25] Crespin A, Le Bescop C, de Gunzburg J, et al. A systematic review and meta-analysis evaluating the impact of antibiotic use on the clinical outcomes of cancer patients treated with immune checkpoint inhibitors. Front Oncol. 2023;13(March):1–21. 10.3389/fonc.2023.1075593. 10.3389/fonc.2023.1075593 PMC10019357 36937417

[26] Pinato DJ, Howlett S, Ottaviani D, et al. Association of prior antibiotic treatment with survival and response to immune checkpoint inhibitor therapy in patients with cancer. JAMA Oncol. 2019;5(12):1774–8. 10.1001/jamaoncol.2019.2785. 31513236 10.1001/jamaoncol.2019.2785 PMC6743060

[27] Hopkins AM, Kichenadasse G, Karapetis CS, Rowland A, Sorich MJ. Concomitant antibiotic use and survival in urothelial carcinoma treated with atezolizumab. Eur Urol. 2020;78(4):540–3. 10.1016/j.eururo.2020.06.061. 32660748 10.1016/j.eururo.2020.06.061

[28] Tinsley N, Zhou C, Tan G, et al. Cumulative antibiotic use significantly decreases efficacy of checkpoint inhibitors in patients with advanced cancer. Oncologist. 2020;25(1):55–63. 10.1634/theoncologist.2019-0160. 31292268 10.1634/theoncologist.2019-0160 PMC6964118

[29] Ahmed J, Kumar A, Parikh K, et al. Use of broad-spectrum antibiotics impacts outcome in patients treated with immune checkpoint inhibitors. Oncoimmunology. 2018;7(11):1–6. 10.1080/2162402X.2018.1507670. 10.1080/2162402X.2018.1507670 PMC6205076 30377571

[30] Anand S, Mande SS. Host-microbiome interactions: gut-liver axis and its connection with other organs. NPJ Biofilms Microbiomes. 2022;8(1). 10.1038/s41522-022-00352-6. 10.1038/s41522-022-00352-6 PMC9626460 36319663

[31] Ramirez J, Guarner F, Bustos Fernandez L, Maruy A, Sdepanian VL, Cohen H. Antibiotics as major disruptors of gut microbiota. Front Cell Infect Microbiol. 2020;10(November):1–10. 10.3389/fcimb.2020.572912. 33330122 10.3389/fcimb.2020.572912 PMC7732679

[32] Hertz FB, Budding AE, van der Lugt-Degen M, Savelkoul PH, Løbner-Olesen A, Frimodt-Møller N. Effects of antibiotics on the intestinal microbiota of mice. Antibiotics (Basel). 2020;9(4):191. 10.3390/antibiotics9040191. Published 2020 Apr 17. 32316518 10.3390/antibiotics9040191 PMC7235770

[33] Duan H, Yu L, Tian F, Zhai Q, Fan L, Chen W. Antibiotic-induced gut dysbiosis and barrier disruption and the potential protective strategies. Crit Rev Food Sci Nutr. 2022;62(6):1427–52. 10.1080/10408398.2020.1843396. 33198506 10.1080/10408398.2020.1843396

[34] Kelly SA, Nzakizwanayo J, Rodgers AM, et al. Antibiotic therapy and the gut microbiome: investigating the effect of delivery route on gut pathogens. ACS Infect Dis. 2021;7(5):1283–96. 10.1021/acsinfecdis.1c00081. 33843198 10.1021/acsinfecdis.1c00081 PMC7618120

[35] Pederzoli F, Bandini M, Raggi D, et al. Is there a detrimental effect of antibiotic therapy in patients with muscle-invasive bladder cancer treated with neoadjuvant pembrolizumab? Eur Urol. 2021;80(3):319–22. 10.1016/j.eururo.2021.05.018. 34053782 10.1016/j.eururo.2021.05.018

