# Supplementary material for: Association between antibiotics and treatment efficacy in metastatic urothelial carcinoma patients
Source: BMC Med. 2025 Feb 25;23:117. doi: 10.1186/s12916-024-03786-1 (PMC11863714; doi:10.1186/s12916-024-03786-1)
Supplement: Supplementary file 1 — Additional file 1: Figs. S1 and Table S1-S5. Fig. S1: Final cohort CONSORT Diagram. Table S1: List of first-line systemic therapy and antibiotic regimens. Table S2: Patient characteristics by antibiotic receipt. aLinear model ANOVA**; bPearson’s chi-squared test**. Table S3: Logistic regression to predict antibiotic receipt. aMultivariable logistic regression; ECOG, Eastern Cooperative Oncology Group; ICI, immunotherapy. Table S4: Three-month landmark overall survival with interaction adjusting for time-varying coefficients. ECOG, Eastern Cooperative Oncology Group. Table S5: Three-month landmark progression-free survival with interaction adjusting for time-varying coefficients. ECOG, Eastern Cooperative Oncology Group. [file 12916_2024_3786_MOESM1_ESM.docx]

**ADITIONAL FILE 1 LEGEND:**

Figure S1: Final Cohort CONSORT Diagram

Table S1: List of First-Line Systemic Therapy and Antibiotic Regimens

Table S2: Patient Characteristics by Antibiotic Receipt

Table S3: Logistic Regression to predict Antibiotic Receipt

Table S4: Three-month Landmark Overall Survival with Interaction adjusting for Time-Varying Coefficients

Table S5: Three-month Landmark Progression Free Survival with Interaction adjusting for Time-Varying Coefficients

**Figure S1:**

**
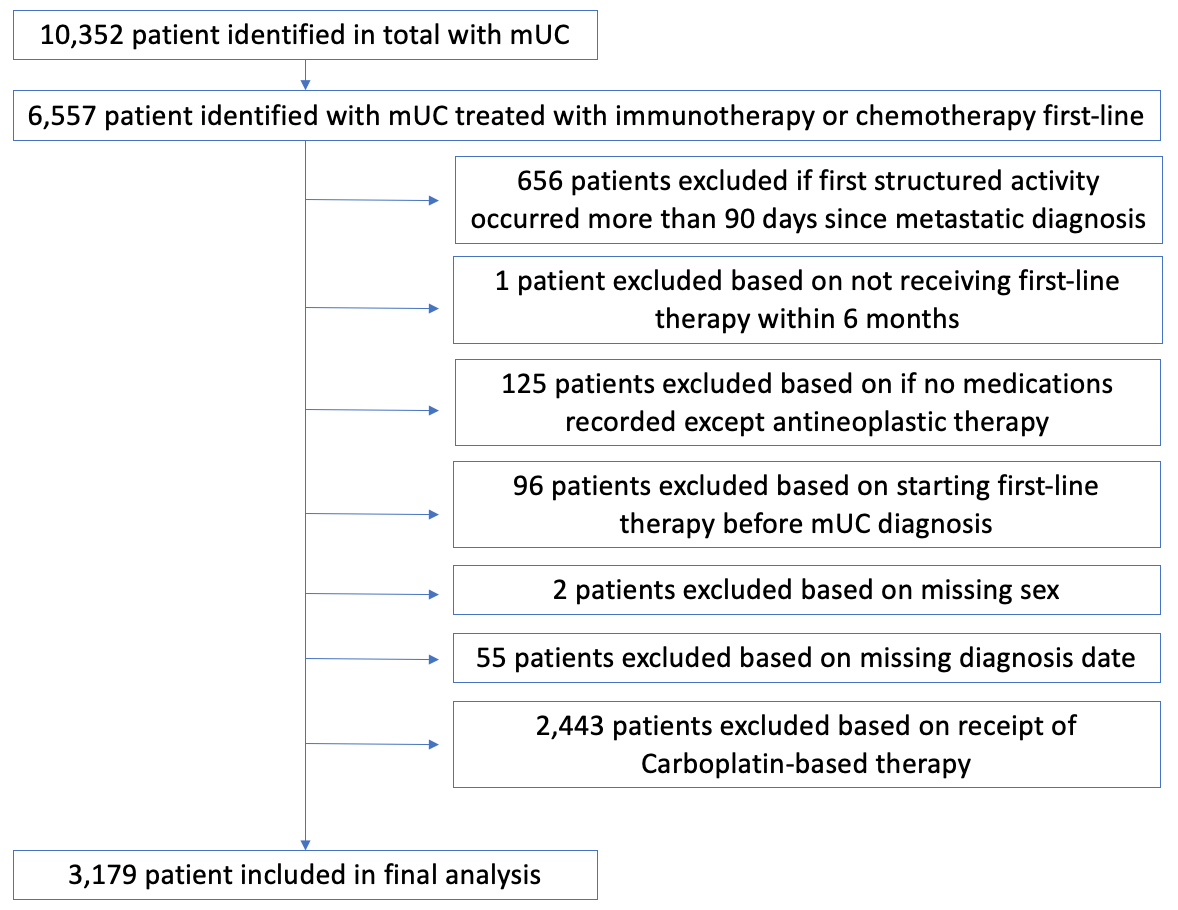
**

**Table S1:**

| **Immunotherapy Regimen** | **N=1483 (%)** |
| --- | --- |
| Atezolizumab  Atezolizumab single agent  Atezolizumab + additional agent(s)  Atezolizumab + chemotherapy  Atezolizumab + TKI | 496 (33.4)  2 (0.1)  31 (2.1)  2 (0.1) |
| Avelumab | 10 (.7) |
| Durvalumab  Durvalumab single agent | 7 (0.5) |
| Nivolumab  Nivolumab single agent  Nivolumab + chemotherapy  Nivolumab + TKI  Nivolumab + Ipilimumab | 115 (7.8)  3 (0.2)  2 (0.1)  2 (0.1) |
| Pembrolizumab  Pembrolizumab single agent  Pembrolizumab + additional agent(s)  Pembrolizumab + chemotherapy  Pembrolizumab + TKI  Pembrolizumab + Atezolizumab | 760 (51.2)  17 (1.1)  28 (1.9)  5 (0.3)  3 (0.2) |
| **Chemotherapy Regimen** | **N=1696 (%)** |
| Cisplatin  Cisplatin single agent  Cisplatin + Gemcitabine  Cisplatin + chemotherapy agent(s)  Cisplatin + *additional agent(s) | 104 (6.1)  1498 (88.3)  88 (5.2)  6 (0.4) |
| **Antibiotic Class**  Cephalosporin  Fluoroquinolone  Glycylcycline  Macrolide  Nitrofuran  Nitroimidazole  Penicillin  Sulfonamide  Tetracycline  Miscellaneous  Mixed | **N=1057 (%)**  158 (14.9)  507 (48)  12 (1.1)  42 (4)  43 (4.1)  11 (1)  85 (8)  83 (7.9)  27 (2.6)  12 (1.1)  77 (7.3) |

**Table S2:**

|  | No (N=2122) | Yes (N=1057) | Total (N=3179) | P value |
| --- | --- | --- | --- | --- |
| Age at Diagnosis |  |  |  | < 0.001^a^ |
| Mean (SD) | 71.4 (9.1) | 69.4 (9.4) | 70.7 (9.2) |  |
| Median | 72.6 | 70.4 | 71.7 |  |
| Range | 31.9-85.1 | 31.6-85.0 | 31.6-85.1 |  |
| Q1, Q3 | 65.2, 79.4 | 63.6, 76.4 | 64.8, 78.5 |  |
| Sex (%) |  |  |  | 0.005^b^ |
| Female | 554 (26) | 326 (31) | 880 (28) |  |
| Male | 1568 (74) | 731 (69) | 2299 (72) |  |
| Stage at Diagnosis (%) |  |  |  | 0.01^b^ |
| I – III | 431 (20) | 219 (21) | 650 (20) |  |
| IV | 728 (34) | 418 (40) | 1146 (36) |  |
| Unknown/Other | 963 (45) | 420 (39) | 1383 (44) |  |
| Race |  |  |  | <0.001^b^ |
| White | 1444 (68) | 774 (73) | 2218 (70) |  |
| Asian | 28 (1.3) | 15 (1.4) | 43 (1.4) |  |
| Black or African American | 78 (3.7) | 52 (4.9) | 130 (4.1) |  |
| Hispanic or Latino | 80 (3.8) | 35 (3.3) | 115 (3.6) |  |
| Other | 308 (15) | 128 (12) | 436 (14) |  |
| Unknown | 184 (8.7) | 53 (5.3) | 237 (7.5) |  |
| Insurance Type (%) |  |  |  | <0.001^b^ |
| Commercial | 660 (31) | 306 (29) | 966 (30) |  |
| Medicaid | 80 (3.8) | 69 (6.5) | 149 (4.7) |  |
| Medicare | 416 (20) | 253 (24) | 669 (21) |  |
| Medicare/Commercial | 503 (24) | 235 (22) | 738 (23) |  |
| Other | 227 (11) | 107 (10) | 334 (10) |  |
| Unknown | 236 (11) | 87 (8.2) | 323 (10) |  |
| Surgery after Diagnosis |  |  |  | 0.1^b^ |
| No | 1847 (87) | 899 (85) | 2746 (86) |  |
| Yes | 275 (13) | 158 (15) | 433 (14) |  |
| ECOG Status (%) |  |  |  | <0.001^b^ |
| 0 | 617 (29) | 288 (27) | 905 (29) |  |
| 1 | 686 (32) | 312 (30) | 998 (31) |  |
| 2 | 219 (10) | 92 (8.7) | 311 (9.8) |  |
| 3 | 108 (2.9) | 28 (2.6) | 96 (3.0) |  |
| 4 | 5 (0.1) | 2 (0.2) | 2 (0.1) |  |
| Unknown | 1105 (30) | 335 (31) | 867 (27) |  |
| Metastasis within 1 month of Diagnosis (%) |  |  |  | 0.2^b^ |
| No | 1195 (56) | 538 (51) | 1733 (54) |  |
| Yes | 927 (44) | 519 (49) | 2573 (46) |  |

**Table 3:**

|  | Odds Ratio | Lower Confidence Interval | Upper Confidence Interval | P value^a^ |
| --- | --- | --- | --- | --- |
| ICI therapy  No  Yes | Ref  0.65 | Ref  0.54 | Ref  0.78 | <0.001 |
| Age at Diagnosis (per 10 years) | 0.81 | 0.73 | 0.89 | <0.001 |
| Body Mass Index | 1.00 | 0.99 | 1.01 | 1 |
| Sex  Female  Male | Ref  0.78 | Ref  0.66 | Ref  0.93 | 0.005 |
| Surgery after Diagnosis  No  Yes | Ref  1.00 | Ref  0.78 | Ref  1.27 | 0.9 |
| Race  White  Hispanic  Asian  Black  Other  Unknown | Ref  0.74  0.95  1.12  0.81  0.54 | Ref  0.48  0.49  0.76  0.64  0.39 | Ref  1.14  1.84  1.65  1.02  0.75 | Ref  0.2  0.9  0.6  0.08  <0.001 |
| Insurance Type  Commercial  Medicaid  Medicare  Medicare/Commercial  Other  Unknown | Ref  1.81  1.65  1.29  1.09  0.78 | Ref  1.25  1.32  1.03  0.83  0.58 | Ref  2.62  2.06  1.61  1.44  1.05 | Ref  0.002  <0.001  0.03  0.5  0.1 |
| Stage at Diagnosis  0  I  II  III  IV  Unknown/Other | Ref  1.25  0.67  1.07  1.08  0.72 | Ref  0.31  0.18  0.29  0.29  0.20 | Ref  5.08  2.51  4.03  4.10  2.62 | Ref  0.8  0.6  0.9  0.9  0.6 |
| Metastatic within 1 month of Diagnosis | 0.77 | 0.56 | 1.05 | 0.102 |
| ECOG Status  0  1  2  3/4  Unknown | Ref  1.07  1.13  1.31  1.43 | Ref  0.87  0.84  0.80  1.17 | Ref  1.31  1.52  2.14  1.76 | Ref  0.5  0.4  0.3  <0.001 |
| Smoking History  No  Yes | Ref  0.91 | Ref  0.76 | Ref  1.09 | 0.3 |

**Table 4:**

|  | Hazard Ratio | Lower Confidence Interval | Upper Confidence Interval | P value |
| --- | --- | --- | --- | --- |
| Age at Diagnosis (per 10 years) | 1.09 | 1.02 | 1.17 | 0.01 |
| Sex  Female  Male | Ref  1.01 | Ref  0.90 | Ref  1.13 | 0.9 |
| Surgery  No  Yes | Ref  0.65 | Ref  0.55 | Ref  0.76 | Ref  <0.001 |
| Body Mass Index | 1.00 | 0.99 | 1.01 | 0.7 |
| Smoking Status  No  Yes | Ref  1.14 | Ref  1.01 | Ref  1.28 | 0.04 |
| Race  White  Hispanic  Asian  Black  Other  Unknown | Ref  0.59  0.63  0.95  0.86  0.97 | Ref  0.42  0.39  0.73  0.73  0.77 | Ref  0.82  1.03  1.23  1.01  1.21 | Ref  0.002  0.06  0.7  0.06  0.8 |
| Stage at Diagnosis  0  I  II  III  IV  Unknown/Other | Ref  0.88  0.83  0.90  1.33  1.14 | Ref  0.33  0.34  0.36  0.55  0.47 | Ref  2.34  2.04  2.21  3.24  2.76 | Ref  0.8  0.7  0.8  0.5  0.8 |
| Insurance Type  Commercial  Medicaid  Medicare  Medicare/Commercial  Other  Unknown | Ref  1.23  0.85  0.96  0.93  1.05 | Ref  0.95  0.73  0.83  0.77  0.88 | Ref  1.59  1.00  1.12  1.13  1.25 | Ref  0.1  0.04  0.6  0.5  0.6 |
| ECOG Status  0  1  2  3/4  Unknown | Ref  1.29  1.53  2.32  1.11 | Ref  1.13  1.25  1.67  0.97 | Ref  1.47  1.88  3.21  1.27 | Ref  <0.001  <0.001  <0.001  0.1 |

**Table 5:**

|  | Hazard Ratio | Lower Confidence Interval | Upper Confidence Interval | P value |
| --- | --- | --- | --- | --- |
| Age at Diagnosis (per 10 years) | 1.02 | 0.96 | 1.10 | 0.5 |
| Sex  Female  Male | Ref  0.99 | Ref  0.88 | Ref  1.11 | 0.8 |
| Surgery  No  Yes | Ref  0.66 | Ref  0.57 | Ref  0.77 | <0.001 |
| Body Mass Index | 1.00 | 0.99 | 1.01 | 0.9 |
| Smoking Status  No  Yes | Ref  0.98 | Ref  0.87 | Ref  1.11 | 0.8 |
| Race  White  Hispanic  Asian  Black  Other  Unknown | Ref  0.90  0.95  1.16  0.89  0.91 | Ref  0.68  0.63  0.91  0.76  0.72 | Ref  1.19  1.45  1.49  1.04  1.14 | Ref  0.4  0.8  0.2  0.2  0.4 |
| Stage at Diagnosis  0  I  II  III  IV  Unknown | Ref  1.23  1.07  1.02  1.59  1.41 | Ref  0.37  0.34  0.32  0.50  0.45 | Ref  4.12  3.40  3.24  4.98  4.43 | Ref  0.7  0.9  0.9  0.4  0.6 |
| Insurance Type  Commercial  Medicaid  Medicare  Medicare/Commercial  Other  Unknown | Ref  0.90  0.92  0.92  0.96  0.96 | Ref  0.68  0.79  0.79  0.79  0.80 | Ref  1.19  1.07  1.07  1.16  1.15 | Ref  0.5  0.3  0.3  0.6  0.6 |
| ECOG Status  0  1  2  3/ 4  Unknown | Ref  1.09  1.42  1.92  1.03 | Ref  0.95  1.15  1.34  0.90 | Ref  1.25  1.76  2.77  1.18 | Ref  0.2  0.001  <0.001  0.7 |
